# Supplementary material for: Prognostic value of baseline [18F]-fluorodeoxyglucose positron emission tomography parameters MTV, TLG and asphericity in an international multicenter cohort of nasopharyngeal carcinoma patients
Source: PLoS One. 2020 Jul 30;15(7):e0236841. doi: 10.1371/journal.pone.0236841 (PMC7392321; doi:10.1371/journal.pone.0236841)
Supplement: S1 Table — (DOCX) [file pone.0236841.s001.docx]

| **Parameters** | **Spearman’s rho** | **p value** |
| --- | --- | --- |
| MTV vs TLG | 0.95 | **<0.001** |
| MTV vs SUV_max_ | 0.54 | **<0.001** |
| MTV vs ASP | 0.35 | **<0.001** |
| TLG vs SUV_max_ | 0.74 | **<0.001** |
| TLG vs ASP | 0.28 | **0.003** |
| SUV_max_ vs ASP | 0.12 | 0.2 |

**S1 table:** Correlation of PET parameters
