# Supplementary material for: Prognostic value of baseline [18F]-fluorodeoxyglucose positron emission tomography parameters MTV, TLG and asphericity in an international multicenter cohort of nasopharyngeal carcinoma patients
Source: PLoS One. 2020 Jul 30;15(7):e0236841. doi: 10.1371/journal.pone.0236841 (PMC7392321; doi:10.1371/journal.pone.0236841)
Supplement: S2 Table — (DOCX) [file pone.0236841.s002.docx]

| **Parameter** | **min. cutoff** | **HR** | **max. cutoff** | **HR** |
| --- | --- | --- | --- | --- |
| **EFS** | | | |  |
| MTV | 7.1 ml | 1.7 | 17.9 ml | 1.9 |
| ASP | 28.5% | 1.7 | 32.6% | 1.7 |
| **OS** | | | |  |
| MTV | 7.1 ml | 1.9 | 24.4 ml | 3.9 |
| ASP | 12% | 2.7 | 16.9% | 2.0 |
| **LRC** | | | |  |
| MTV | 8.4 ml | 2.0 | 24.4 ml | 4.8 |

**S2 table:** Minimum and maximum cutoff values of PET parameters, leading at least to a trend for significance (p≤0.1) upon univariate testing.
