# Supplementary material for: Prognostic value of baseline [18F]-fluorodeoxyglucose positron emission tomography parameters MTV, TLG and asphericity in an international multicenter cohort of nasopharyngeal carcinoma patients
Source: PLoS One. 2020 Jul 30;15(7):e0236841. doi: 10.1371/journal.pone.0236841 (PMC7392321; doi:10.1371/journal.pone.0236841)
Supplement: S5 Fig — Kaplan-Meier curves of the PET parameter ASP in the Chinese exploration cohort (a) and all other centers as validation cohort (b) with respect to OS. (DOCX) [file pone.0236841.s007.docx]

**S7 figure:** Kaplan-Meier curves of the PET parameter ASP in the Chinese exploration cohort (a) and all other centers as validation cohort (b) with respect to OS.
