# Supplementary material for: Prognostic value of baseline [18F]-fluorodeoxyglucose positron emission tomography parameters MTV, TLG and asphericity in an international multicenter cohort of nasopharyngeal carcinoma patients
Source: PLoS One. 2020 Jul 30;15(7):e0236841. doi: 10.1371/journal.pone.0236841 (PMC7392321; doi:10.1371/journal.pone.0236841)
Supplement: Attachment — Submitted filename: response to reviewers.docx [file pone.0236841.s008.docx]

PONE-D-20-13504
Prognostic value of baseline [18F]-fluorodeoxyglucose positron emission tomography parameters MTV, TLG and asphericity in an international multicenter cohort of nasopharyngeal carcinoma patients
PLOS ONE

Dear Dr. Sebastian Zschaeck,

Thank you for submitting your manuscript to PLOS ONE. After careful consideration, we feel that it has merit but does not fully meet PLOS ONE’s publication criteria as it currently stands. Therefore, we invite you to submit a revised version of the manuscript that addresses the points raised during the review process.

==============================
Please submit your revised manuscript by 20 June 2020. If you will need more time than this to complete your revisions, please reply to this message or contact the journal office at [plosone@plos.org](mailto:plosone@plos.org). When you're ready to submit your revision, log on to <https://www.editorialmanager.com/pone/> and select the 'Submissions Needing Revision' folder to locate your manuscript file.

Please include the following items when submitting your revised manuscript:

- A rebuttal letter that responds to each point raised by the academic editor and reviewer(s). You should upload this letter as a separate file labeled 'Response to Reviewers'.
- A marked-up copy of your manuscript that highlights changes made to the original version. You should upload this as a separate file labeled 'Revised Manuscript with Track Changes'.
- An unmarked version of your revised paper without tracked changes. You should upload this as a separate file labeled 'Manuscript'.

If you would like to make changes to your financial disclosure, please include your updated statement in your cover letter. Guidelines for resubmitting your figure files are available below the reviewer comments at the end of this letter.

If applicable, we recommend that you deposit your laboratory protocols in protocols.io to enhance the reproducibility of your results. Protocols.io assigns your protocol its own identifier (DOI) so that it can be cited independently in the future. For instructions see: <http://journals.plos.org/plosone/s/submission-guidelines#loc-laboratory-protocols>

We look forward to receiving your revised manuscript.

Kind regards,

Domenico Albano
Academic Editor
PLOS ONE

Journal Requirements:

When submitting your revision, we need you to address these additional requirements.

1. Please ensure that your manuscript meets PLOS ONE's style requirements, including those for file naming. The PLOS ONE style templates can be found at

<https://journals.plos.org/plosone/s/file?id=wjVg/PLOSOne_formatting_sample_main_body.pdf> and

<https://journals.plos.org/plosone/s/file?id=ba62/PLOSOne_formatting_sample_title_authors_affiliations.pdf>

*We changed the formatting accordingly*

2. We note that you have indicated that data from this study are available upon request. PLOS only allows data to be available upon request if there are legal or ethical restrictions on sharing data publicly. For information on unacceptable data access restrictions, please see <http://journals.plos.org/plosone/s/data-availability#loc-unacceptable-data-access-restrictions>.

In your revised cover letter, please address the following prompts:

a) If there are ethical or legal restrictions on sharing a de-identified data set, please explain them in detail (e.g., data contain potentially identifying or sensitive patient information) and who has imposed them (e.g., an ethics committee). Please also provide contact information for a data access committee, ethics committee, or other institutional body to which data requests may be sent.

b) If there are no restrictions, please upload the minimal anonymized data set necessary to replicate your study findings as either Supporting Information files or to a stable, public repository and provide us with the relevant URLs, DOIs, or accession numbers. Please see <http://www.bmj.com/content/340/bmj.c181.long> for guidelines on how to de-identify and prepare clinical data for publication. For a list of acceptable repositories, please see <http://journals.plos.org/plosone/s/data-availability#loc-recommended-repositories>.

We will update your Data Availability statement on your behalf to reflect the information you provide.

*Original PET data upload, even in pseudonymized form is restricted by German data protection law. We have changed the corresponding author, since Qin Lin has all original data and should be contacted in case of any requests:*

*Qin Lin*

*Xiamen Cancer Hospital, The First Affiliated Hospital of Xiamen University, Xiamen, China*

*No. 55 Zhenhai Road, Siming District, Xiamen City, Fujian Province, China, 361003*

*Tel: +86-0592-2137256*

*Fax: +86-0592-2137189*

*Email: linqin05@163.com*

3. Thank you for stating the following in the Competing Interests section:

"Dr. Amthauer reports personal fees from SIRTEX Medical Europe, grants from GE Healthcare, grants and personal fees from Novartis, outside the submitted work;
All other authors have declared that no competing interests exist."

Please confirm that this does not alter your adherence to all PLOS ONE policies on sharing data and materials, by including the following statement: "This does not alter our adherence to  PLOS ONE policies on sharing data and materials.” (as detailed online in our guide for authors [http://journals.plos.org/plosone/s/competing-interests](about:blank)).  If there are restrictions on sharing of data and/or materials, please state these. Please note that we cannot proceed with consideration of your article until this information has been declared.

Please include your updated Competing Interests statement in your cover letter; we will change the online submission form on your behalf.

*We have included the updated statement in the cover letter.*

Please know it is PLOS ONE policy for corresponding authors to declare, on behalf of all authors, all potential competing interests for the purposes of transparency. PLOS defines a competing interest as anything that interferes with, or could reasonably be perceived as interfering with, the full and objective presentation, peer review, editorial decision-making, or publication of research or non-research articles submitted to one of the journals. Competing interests can be financial or non-financial, professional, or personal. Competing interests can arise in relationship to an organization or another person. Please follow this link to our website for more details on competing interests: [http://journals.plos.org/plosone/s/competing-interests](about:blank)

Additional Editor Comments (if provided):

I believe that the topic of the paper is interesting and of clinical interest. There are some points to clarify and discuss following reviewers suggestions.

*I hope that we have been able to address all questions and suggestions raised by the	reviewers.*


Reviewers' comments:

Reviewer's Responses to Questions

**Comments to the Author**

1. Is the manuscript technically sound, and do the data support the conclusions?

The manuscript must describe a technically sound piece of scientific research with data that supports the conclusions. Experiments must have been conducted rigorously, with appropriate controls, replication, and sample sizes. The conclusions must be drawn appropriately based on the data presented.

Reviewer #1: Yes

Reviewer #2: Yes

Reviewer #3: Partly

2. Has the statistical analysis been performed appropriately and rigorously?

Reviewer #1: Yes

Reviewer #2: Yes

Reviewer #3: No

3. Have the authors made all data underlying the findings in their manuscript fully available?

The [PLOS Data policy](http://www.plosone.org/static/policies.action#sharing) requires authors to make all data underlying the findings described in their manuscript fully available without restriction, with rare exception (please refer to the Data Availability Statement in the manuscript PDF file). The data should be provided as part of the manuscript or its supporting information, or deposited to a public repository. For example, in addition to summary statistics, the data points behind means, medians and variance measures should be available. If there are restrictions on publicly sharing data—e.g. participant privacy or use of data from a third party—those must be specified.

Reviewer #1: No

Reviewer #2: Yes

Reviewer #3: No

4. Is the manuscript presented in an intelligible fashion and written in standard English?

PLOS ONE does not copyedit accepted manuscripts, so the language in submitted articles must be clear, correct, and unambiguous. Any typographical or grammatical errors should be corrected at revision, so please note any specific errors here.

Reviewer #1: Yes

Reviewer #2: Yes

Reviewer #3: Yes

5. Review Comments to the Author

Please use the space provided to explain your answers to the questions above. You may also include additional comments for the author, including concerns about dual publication, research ethics, or publication ethics. (Please upload your review as an attachment if it exceeds 20,000 characters)

Reviewer #1: The authors present a retrospective analysis of different datasets – one from their own institution (24 patients), one form a published cohort from China (57 patients) and one from an open source dataset from Canada (26 patients).
The aim was to investigate the best predictive value on FDG PETCT for OS, PFS and local control for head and neck cancer prior to curative radio or radio-chemotherapy.

General comment: This is a well written study incorporating PET parameters and asphericity to predict outcome. It is an interesting concept to use open source data to increase the cohort especially in relatively rare entities. It would increase the value of the presented work if a clear separation between test and validation cohort would be performed. As the authors stated correctly – the association of MTV or TLG with poor prognosis has been investigated in numerous mono-center studies. Scanner and protocol differences have a high impact on quantitative PET parameters – the question whether there is a robust cut off, allowing to predict outcome for NPC cancer is of interest. However, the way it is performed in the current study this is more of a collection in multiple centers with a single center analysis and not really a multicenter analysis. One could think of taking the cohort from Berlin and China to generate the cut offs and use Canada as a validation group.

*We thank the reviewer for these valuable remarks. We included additional patients	from public available datasets and split the patients into exploration and validation	cohort (due to the low number of patients in all other cohorts we used only the	Chinese cohort for establishment of cut-off values and all other centers for	validation). By this approach we were able to validate the high prognostic value of	MTV for all endpoints (see results and supplementary figures 4-7), regardless of	differences between scanners/ geographic location. We thank the reviewer for this	suggestion, as we think this further strengthens the value of the manuscript.	Nonetheless due to the relatively low number of patients we additionally kept the	original cut-off values for the whole cohort within the manuscript (although they are	very close to the cut-off values optimized for Chinese patients).*

With four outcome parameters you should think about correction of significance by a factor of 4: or you reduce the parameters to PFS and OS.

*We agree that in case of multiple testing like in the current study a Bonferroni	correction is necessary for a final assessment of the performance of the parameters.	However, this study has to be considered as explorative and for a final assessment	further investigations are necessary. Therefore, we omitted this correction.*

Specific comments:
Abstract Methods:
How did you generate the binarized cutoff values? A brief statement in the methods should be available.
How was (MTV + ASP) generated – either value above a certain cut off – same cut off used for the evaluation of MTV and ASP alone?

*We added both information*

Intro:
did you look into the EBV distribution in your cohort? Was there a difference in FDG uptake between EBV + / - cases? (It was suggested that HPV+ HNSCC have less FDG uptake the HPV- tumors).

*There is no significant difference in FDG uptake between EBV +/- cases. However,	there are only 6 EBV- cases and, therefore, these results are not very conclusive.	We would prefer not to show them due to the low number of patients and limited	validity, but if the reviewer insists we will add these results.*

Methods:
Patients – you should state how many patients were collected where – not just in table one – but in the patients section – ideally also declaring the test and validation cohort.

*We have added the information as suggested.*

The range of injected activity from 132-770 is rather wide – where there body weight adapted injection protocols?

*The injected dose was weight adapted in the German data and not weight adapted in	the Chinese data. For the American data information on the injection protocol was	not available. All data with very high injected dose were from America and these	scans had a relative large uptake time (>100min) and the corresponding patients had	a large weight (>100Kg). This suggests that the injected dose was at least partly	weight and uptake time adapted, but this is just speculation.*

Segmentation and time correction are done very carefully.
The definition of MTV+ASP is missing. Why “and” – did you also investigate “or”?

*We have added the definition of MTV+ASP in the abstract and in the methods section.	Regarding ‘and’ vs. ‘or’: the clinical goal in the current patient group is a therapy	intensification for the patients with high risk. To justify this, the false positive rate of	the outcome prediction must be low, which can be achieved by an ‘and’ combination	of the risk factors. An ‘or’ combination would improve the false negative rate. That’s	why we investigated only the ‘and’ combination. We now also analyzed the ‘or’	combination: it is a significant predictor of OS, but it is not independent from MTV	and ASP alone. This makes sense as nasopharyngeal carcinomas have a relatively	good outcome and there are only few events for the investigated endpoints. We prefer	not the add this to the manuscript, but if the reviewer	insists we will add a	comment on that.*

Results:
Tables should be accompanied with the abbreviation explanation.
It seems that indeed EBV+ tumors have a better outcome – the association with lower FDG uptake therefore seems very probable. I would suggest adding this to the results, despite the small number of EBV-.

Your data suggests that MTV is good for OS and TLG for LRC – but both have a correlation of 0.95 – don’t you think that this rather reflects coincidence within your cohort than a biological difference between the meaning of TLG and MTV?

*We have added the abbrevioants, that have not been introduced as suggested.*

*We fully agree with the reviewer that MTV and TLG show a very high correlation and	both parameters probably have very similiar prognostic value. Given the slightly	diffferent results with the additionally included patients, MTV is now prognostic for	all endpoints. Therefore, we decided to focus on MTV and think that TLG is pretty	redundant in this setting.*

Discussion:
How can you rule out distant disease for NPC patients if your field of view did not cover the aorta?

*We express our respect for this very smart comment! In fact most of the patients	received their FDG-PET examinations for radiation treatment planning. This means	that the PET scan is performed with thermoplastic mask in the head and neck region.	For patient comfort the rest of the body is scanned without mask. Therefore usually	the PET scans are split into two different series (which can for example be observed	in the dataset of the MD Anderson cohort). Unfortunately this hampers analysis of the	aorta and the tumor region at the same time. We added a short comment on this.*

You suggest that a PET based treatment individualization should demonstrate some benefits – However you do not specify how you think this should be done – should patient with a TLG above 130 get higher doses, or more safety margins?

*We changed the discussion according to the new findings and the	reviewers´suggestions and would now prefer MTV for treatment individualization. As	discussed by the publication from Liu and colleagues we think that dose escalation	within high-uptake FDG sub-volumes might be a promising aproach. We added a	remark on this topic.*

Reviewer #2: Thank you to review this interesting paper.

The study investigated the prognostic value of FDG-PET in a large patient cohort of nasopharyngeal cancers. The involved patients were from Centers of Germany (24 patients), China(57 patients), and Canada (26 patients).

The authors used established PET parameters, comprising SUV, MTV, TLG and also a novel asphericity parameter.

MTV and asphericity were significantly associated with overall survival.
There is definite need to evaluate novel FDG-PET parameters in a multicenter setting.

The imaging analysis and statistical analysis is profound and the results promising.

The manuscript is well written and the conclusion drawn is reasonable.

I don't have concerns regarding this manuscript.

*We thank the reviewer very much for his kind and motivating remarks*

Reviewer #3: 1. The authors state that most of the data on prognostic impact of metabolic parameters from pre-treatment FDG-PET/CT in nasopharyngeal cancers comes from the endemic Chinese (Asian) population and hence they intended to study it in a multi-centre non-Asian cohort from Europe and America. Unfortunately over half the patients (n=57) in this study cohort are from China itself which defeats the purpose completely. It is recommended that this analysis be repeated after removing the Chinese patient cohort.

*We thank the reviewer for this comment. We hope that we addressed this issue by	using Chinese patients for generation of cut-off values and validating these cut-offs in	European/ American patients. By this approach both cohorts are evaluated	separately.*

2. The authors acknowledge that two previous systematic reviews and meta-analysis have established the prognostic importance of SUXmax, MTV, and TLG in nasopharynx, but go on to state that the Canadian and Chinese cohort that has been included in the present analysis were not part of those reviews. Both the Canadian (ref 13, 2012) and Chinese (ref 14, 2013) studies were published well before the meta-analysis (2017), so it is indeed surprising that neither of the two meta-analysis included those data.

*We agree that this might be surprising. The non-inclusion of the Canadian (and the	other patients now additionally included) in the meta-analyses is due to the fact, that	these patients were reported together with head and neck carcinomas of other sites.	Therefore, the authors of the meta-analyses were not able to extract data of	nasopharyngeal patients out of these publications. Regarding the publication of	Chinese patients: The publication describes interim PET findings but does not include	any oncological outcome parameters, therefore it was not included in the meta-	analyses.*

3. The authors have used multi-variate Cox regression modelling for identifying prognostic factors for LRC, DMFS, EFS, and OS. However, many of the metabolic PET parameters display collinearity (SUVmax, MTV, TLG) which complicates such analysis. It is not entirely clear from the statistical section, whether collinearity was assessed and/or addressed in the study.

*We fully agree with reviewer, that due to collinearity not all parameters can be tested	in multivariate analysis. That’s why we excluded TLG from this analysis (high	correlation with MTV). We have added this information to the abstract. All	correlations are listed in a table in the supplement.*

4. There is no biological explanation for finding TLG significant for LRC and MTV/ASP for OS. Even more intriguing is the fact that they did not find association of DMFS with any of the metabolic PET parameters.

*Given the additionally included patients, MTV is now prognostic for all endpoints	except FFDM. We think this is in-line with recent publications, as lymph-node	parameters might be more closely related to the development to distant metastases (as	suggested by the quoted studies from Xiao and Jin and taking into account a common	mechanistical model in which most tumors develop the ability to metastazize to	lymphnodes before developing distant metastases).*

*Regarding the biological explanation, we kindly disagree. There are preclinical	experiments suggesting a role of high FDG uptake and radioresistance (just one	example for a very thoroughly designed in vivo study: Impact of Pre- And Early Per-	Treatment FDG-PET Based Dose-Escalation on Local Tumour Control in	Fractionated Irradiated FaDu Xenograft Tumours by Christina Jentsch and	colleagues). Additionally it is known that volume (which is part of MTV) is related to	a decrease of local control by radiotherapy. ASP was chosen as a parameter because	it can reflect tumor necrosis. As tumor necrosis is associated with higher	proliferation	and intratumoral hypoxia (both known adverse biological factors), we think that all	investigated parameters have a clear biological rationale.*

5. ASP is a crude measure of tumor heterogeneity which can be measured in a more refined manner using modern radiomics approach (several open source software programs allows more refined assessment and analysis of tumor heterogeneity).

*We agree that there are many more heterogeneity parameters. However, the goal was	to explicitely study ASP, which showed very stable results in other investigation,	including no- nasopharyngeal head and neck cancers. The results of the present study	show, that this is also the case in the current patient group, and, therefore provide	further evidence that APS is robust predictor for therapy outcome. Including more	heterogeneity parameters would hide the actual goal.*

6. The authors have reported the follow-up of only surviving patients, they should report the follow-up of the entire study cohort including the inter-quartile range.

*We have added the information as suggested.*
